# Where Are We? The Past, Present and Future of Thoracic Ultrasound

**DOI:** 10.3390/jcm12144559

**Published:** 2023-07-08

**Authors:** Alessandro Zanforlin

**Affiliations:** Service of Pulmonology, Health District of Bolzano (SABES-ASDAA), Lehrkrankenhaus der Paracelsus Medizinischen Privatuniversität, 39100 Bolzano-Bozen, Italy; alessandro.zanforlin@gmail.com

The technique of thoracic ultrasound is living through a progressive rise in clinical routine.

After initial skepticism due to the impossibility of studying lung parenchyma with the available ultrasound machines, the increasing evidence and the growth of ultrasound technology have permitted the development of ultrasound use in every application in respiratory care.

In pleural diseases, ultrasound has become the gold standard in the management of pleural effusions, as a diagnostic tool and as a guide for interventional procedures [1].

The study of diaphragmatic features and motions, after the evaluation of normal features [2], is being characterized for use in muscle dysfunctions in neuromuscular diseases [3] and in intensive care settings, being a tool for predicting outcomes of critical patients [4].

The ultrasound assessment of lung diseases is continuously growing; today, lung ultrasound is a fundamental tool to diagnose and follow-up pneumonia [5] and has a promising role in the early detection of interstitial lung diseases [6].

However, the technique has limits, given the impossibility of ultrasound waves to cross the ventilated (even partially) peripheral air spaces.

From a search on Pubmed for the terms “thoracic ultrasound”, “chest ultrasound”, “lung ultrasound”, “pleura ultrasound”, and “diaphragm ultrasound” we obtained the number of papers written yearly since 1945. This number is progressively increasing, reaching a huge peak of more than 21,000 total papers in 2021 and 2022, thanks in part to COVID-19-related research (Figure 1).

This literature jungle has evolved from case reports and descriptions of artifacts and signs for lung study protocols and clinical trials, up to physical models to explain the origin of different vertical artifacts [7] and studies on the possible role of artificial intelligence [8]. In the pandemic era, the role of lung ultrasound was crucial, particularly in acute diagnostic settings and in critical care management [9,10].

So, what do we need today?

Much has been written about lungs and pleura, but the study of the whole respiratory muscular system and the global mechanics of the chest wall have yet to be fully explained and described.

Moreover, all the known notions must be put in order, to finally assess the correct terminology and establish an appropriate and universal methodology. Thoracic ultrasound escapes from the pure “diagnostic imaging” because its features do not permit a precise diagnosis when only considering the obtained images. In fact, it becomes wholly the responsibility of an operator, who needs “good eyes” to correctly interpret the image, “good hands” to adequately guide the probe and create a good quality image, and finally a “good mind”, to understand the clinical situation of the patient and integrate clinical data with the procedure, to obtain a final, accurate diagnosis. 

That is art.

## Figures and Tables

**Figure 1:**
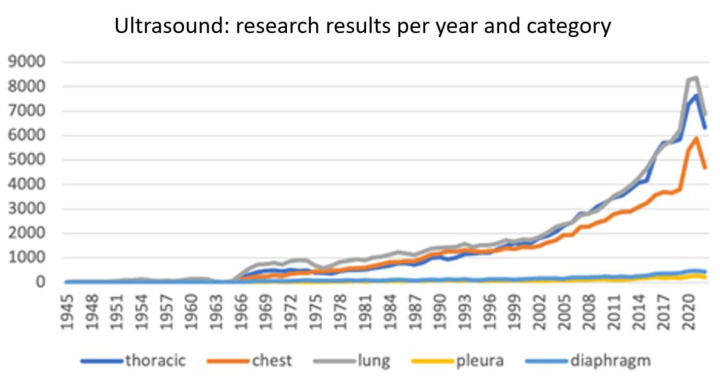
Research results on Pubmed for specific types of ultrasound.
